# Dapagliflozin Attenuates Post-Infarction Myocardial Fibrosis Associated With the Inhibition of NLRP3-Dependent Inflammation

**DOI:** 10.31083/RCM51179

**Published:** 2026-10-20

**Authors:** Xiao Han, Jun Zhang, Pingchang Guo, Junshun Chang, Bing Liu, Xin Diao, Ping Wu, Sheng He, Hua Wei, Wenjuan Dai, Yu Hong, Yuwen Chen, Li Li, Ruonan Wang, Zhifang Wu, Sijin Li

**Affiliations:** ^1^Collaborative Innovation Center for Molecular Imaging of Precision Medicine, Shanxi Medical University, 030000 Taiyuan, Shanxi, China; ^2^Department of Nuclear Medicine, First Hospital of Shanxi Medical University, 030000 Taiyuan, Shanxi, China; ^3^Department of Cardiology, First Hospital of Shanxi Medical University, 030000 Taiyuan, Shanxi, China; ^4^Department of Radiology, First Hospital of Shanxi Medical University, 030000 Taiyuan, Shanxi, China; ^5^Department of Biochemistry and Molecular Biology, College of Basic Medical Sciences, Shanxi Medical University, 030000 Taiyuan, Shanxi, China

**Keywords:** sodium-glucose transporter 2, myocardial infarction, ventricular remodeling, fibroblasts, fibroblast activation protein alpha, NLR family, pyrin domain-containing 3 protein

## Abstract

**Background:**

Sodium–glucose cotransporter 2 (SGLT2) inhibitors have attracted considerable attention owing to their unexpected cardioprotective benefits in addition to glycemic control. Indeed, these inhibitors have been reported to exert anti-fibrotic effects and ameliorate NACHT, leucine-rich repeat (LRR), and pyrin domain-containing protein 3 (NLRP3)-dependent inflammation. However, whether SGLT2 inhibitors (SGLT2is) attenuate post-infarction myocardial fibrosis by suppressing NLRP3-dependent signaling remains unclear. Thus, this study aimed to investigate the mechanisms through which the SGLT2is dapagliflozin (DAPA) mitigates post-infarction myocardial fibrosis, with particular emphasis on the modulation of NLRP3-dependent signaling.

**Methods:**

Male Sprague-Dawley rats were used to establish a myocardial infarction (MI) model via left coronary artery ligation and were treated with DAPA (1 mg/kg/day). Cardiac magnetic resonance imaging was performed to evaluate cardiac function and remodeling. Serial ^18^F-fibroblast activation protein inhibitor-74 (^18^F-FAPI-74) positron emission tomography (PET) was used to track temporal changes in cardiac fibroblast (CF) activation. Myocardial fibrosis and NLRP3-dependent signaling were assessed* ex vivo*. The effects of DAPA on NLRP3-dependent signaling, fibroblast proliferation, phenotypic transition, and collagen synthesis were determined *in vitro* using cardiac fibroblasts (CFs) stimulated with lipopolysaccharide.

**Results:**

Compared with untreated MI rats, MI rats treated with DAPA exhibited significantly lower left ventricular (LV) end-systolic volume, LV end-diastolic volume, and LV mass, along with improved LV ejection fraction (57.6 ± 2.4% vs. 54.4 ± 2.1% in the MI group; *p *= 0.011). LV fibrosis was reduced in DAPA-treated MI rats (20.1 ± 1.9% vs. 31.1 ± 4.0% in the MI group; *p *= 0.0026), and fibrosis markers were significantly downregulated. *In vivo *^18^F-FAPI-74 PET revealed that DAPA suppressed CF activation, as demonstrated by significantly reduced ^18^F-FAPI-74 uptake in the infarcted region. In infarcted cardiac tissue, DAPA inhibited the expression of the NLRP3 inflammasome, interleukin-1 beta (IL-1β), and interleukin-18 (IL-18). CF experiments performed *in vitro* showed that DAPA inhibited the nuclear factor kappa B (NF-κB)/NLRP3 pathway and attenuated CF proliferation, phenotypic transition, and collagen synthesis.

**Conclusions:**

By suppressing CF activation, DAPA improved myocardial fibrosis, adverse LV remodeling, and cardiac function post-MI, in association with inhibition of the NF-κB/NLRP3 pathway. ^18^F-FAPI-74 PET enabled non-invasive visualization of the anti-fibrotic effects of DAPA post-MI and may serve as a tool to monitor anti-fibrotic treatment efficacy after MI.

## 1. Introduction

Acute myocardial infarction remains a major contributor to cardiovascular deaths worldwide and is one of the leading causes of heart failure (HF) [1]. Despite substantial advances in myocardial reperfusion therapy, post-myocardial infarction (MI) HF continues to be prevalent and is associated with poor prognosis [2]. Myocardial fibrosis and adverse left ventricular remodeling (ALVR) are key pathological bases of post-MI HF [3] and are closely associated with the inflammatory cascade following MI. Although moderate inflammation facilitates the clearance of necrotic cardiac tissue debris and promotes wound healing, excessive or sustained inflammation drives cardiac fibroblast (CF) activation and extracellular matrix deposition [4], thereby accelerating ALVR and worsening cardiac function. Thus, targeting inflammation has emerged as a promising strategy for secondary prevention to improve the prognosis after MI [5].

The NACHT, LRR, and PYD domain-containing protein 3 (NLRP3) inflammasome [6] has been widely recognized as a central regulator linking post-infarction inflammation to fibrosis [7]. It activates the effector caspase-1 (Casp-1) and triggers the maturation of interleukin-1 beta (IL-1β) and interleukin-18 (IL-18), which further amplify myocardial inflammation. A previous study reported that the NLRP3 inflammasome is highly up-regulated in cardiac fibroblasts (CFs) after MI and may substantially contribute to infarct size progression during ischaemia-reperfusion injury [8], implicating it as an important therapeutic target for post-infarction myocardial fibrosis. Furthermore, recent preclinical studies showed that the administration of MCC950, a highly specific and well-established NLRP3 inhibitor, effectively attenuated myocardial fibrosis following MI [9,10,11]. These findings highlight the need for further studies to develop therapeutic strategies that target NLRP3-dependent inflammation to limit myocardial fibrosis and ALVR after MI.

Sodium-glucose transporter 2 (SGLT2) inhibitors (SGLT2is) are a novel class of oral hypoglycemic agents that lower blood glucose levels by inhibiting renal glucose reabsorption in an insulin-independent manner. In addition to their glucose-lowering effects, large clinical trials have demonstrated that SGLT2is significantly reduce the risk of hospitalization for HF in both diabetic and non-diabetic populations [12,13]. Experimental studies have shown that SGLT2is can improve cardiac fibrosis and function in several cardiomyopathy models [14,15,16,17,18]. In the myocardial ischemia setting, SGLT2is has been reported to reduce the programmed death of cardiomyocytes and attenuate myocardial fibrosis [19,20,21]. However, the mechanisms underlying their anti-fibrotic effects after MI remain poorly understood and have not been dynamically visualized *in vivo*. Recent evidence indicates that SGLT2is exhibit anti-inflammatory properties [17,22,23,24] and suppresses NLRP3 inflammasome activation [22,25,26]. It has not yet been clarified that SGLT2is attenuate post-infarction fibrosis by inhibiting CF activation through the modulation of NLRP3-dependent inflammation after MI. We therefore hypothesized that SGLT2is mitigate myocardial fibrosis and ALVR by attenuating the maladaptive activation of CFs following MI, mediated via the NLRP3-dependent signaling.

Concurrently, recent advances in fibroblast activation protein (FAP)-targeted molecular imaging [27,28] have enabled the noninvasive visualization of CF activation following MI [29,30]. This is achieved by selectively targeting FAP, which is highly expressed in activated CFs but minimally expressed under quiescent conditions [31]. It is strongly associated with long-term left ventricular (LV) remodeling post-MI and serves as a novel imaging biomarker for the early prediction of post-MI ALVR [32,33]. However, their application in monitoring the efficacy of post-MI anti-fibrotic treatment remains unexplored.

In this context, this study aimed to elucidate the underlying molecular mechanisms by which the SGLT2is dapagliflozin (DAPA) attenuates post-infarction myocardial fibrosis, with an emphasis on the modulation of NLRP3-dependent signaling, using a non-diabetic rat model of MI and lipopolysaccharide (LPS)-stimulated CFs. Furthermore, based on^18^F-fibroblast activation protein inhibitor (FAPI)-74 (^18^F-FAPI-74) positron emission tomography (PET), the anti-fibrotic effects of DAPA after MI were non-invasively and dynamically evaluated by visualizing its effects on CF activation *in vivo*. The utility of ^18^F-FAPI-74 PET for tracking the efficacy of anti-fibrotic treatment was preliminarily explored.

## 2. Materials and Methods

### 2.1 Animals and Induction of MI

A total of sixty healthy male Sprague-Dawley rats (6 weeks old, 200–220 g) were commercially obtained from Beijing Vital River Laboratory Animal Technology Co., Ltd. (China). The rats were housed in a specific pathogen-free facility under standard conditions (temperature: 20–25 ℃, humidity: 60%, 12-hour light/12-hour dark cycle), with three to five rats per housing unit. The rats were acclimated for one week with free access to food (standard rat chow F002, SPF (Beijing) Biotechnology Co., Ltd., Beijing, China) and water. The MI model was established via permanent ligation of the left anterior descending (LAD) coronary artery. Rats were fasted for 12 hours (h) before surgery but had free access to water. Anesthesia was induced with 2% isoflurane (Cat. No. R510-22-10, Shenzhen RWD Life Science Co., Ltd., Shenzhen, China) via inhalation, followed by endotracheal intubation and connection to a small animal ventilator for continuous oxygen supply. Real-time electrocardiographic monitoring was performed throughout the surgery. At the surgical incision site, fibroblast repair activity may result in the accumulation of the ^18^F-FAPI-74 radiotracer in PET imaging. To avoid interference with the myocardial PET images, a median thoracotomy was performed to expose the heart for LAD ligation. The LAD was ligated approximately 2–3 mm from its origin using 7-0 polypropylene sutures (Cat. No. W8704, Ethicon, Somerville, NJ, USA). Successful MI induction was confirmed by pale discoloration or cyanosis of the LV free wall, along with ST segment elevation observed on the electrocardiogram (ECG). Infarct size was visually assessed based on the extent of pale or cyanotic regions of the myocardium, and rats with an infarct size of approximately 30% were included in the study [34]. Sham-operated rats underwent identical thoracotomy and heart manipulation without LAD ligation.

### 2.2 Animals Experimental Design

One week prior to surgery, the rats were randomly assigned in a 1:1 ratio to receive either standard chow or chow supplemented with DAPA (Cat. No. HY-10450, CAS: 461432-26-8, MedChemExpress, Monmouth Junction, NJ, USA). Based on the average food intake and body weight (BW) of the rats, DAPA was blended into standard chow at a concentration of 12 mg/kg, providing an estimated daily dose of approximately 1 mg/kg. Weekly food intake was recorded to confirm comparable DAPA exposure among rats (**Supplementary Table 1**), suggesting similar DAPA intake for each rat.

Preoperative baseline assessments of myocardial viability, CF activation, and cardiac function were performed in ten randomly selected rats per group using ^18^F-fluorodeoxyglucose (^18^F-FDG) PET, ^18^F-FAPI-74 PET, and cardiac magnetic resonance (CMR), respectively. On the day of surgery, all rats were randomized to sham surgery or LAD ligation to induce MI. No deaths occurred in sham-operated rats. Six rats died (two rats in the MI group and four rats in the MID group) within 4 h of the MI-induced surgical procedure because of malignant arrhythmias. Additionally, two rats in the MI group were excluded because their infarct sizes did not meet the predefined inclusion criteria. 52 surviving rats were enrolled for subsequent experiments and divided into four experimental groups: (1) sham-operated rats receiving standard chow (Sham group, n = 12), (2) sham-operated rats receiving standard chow containing DAPA (ShamD group, n = 12), (3) MI rats receiving standard chow (MI group, n = 14), and (4) MI rats receiving standard chow containing DAPA (MID group, n = 14). To evaluate the short-term cardiac benefits of DAPA post-MI (one week after surgery), *in vivo* imaging assessments were performed. Five rats per group were randomly euthanized via inhalation of an overdose of isoflurane, followed by thoracotomy to ensure death for *ex vivo* analysis. CF activation was noninvasively tracked* in vivo.* Following cardiac function assessment, the remaining 32 rats were sacrificed as previously described 42 days after surgery. Throughout the experiment, rats in the same group were housed with food and water available ad libitum, except for surgical and imaging requirements. BW (g), fasting blood glucose (FBG, mmol/L), heart rate (HR, beats per minute, bpm), and blood pressure (BP, mmHg) were monitored.

Randomization was performed using an Excel-generated random number table, and group assignment was concealed from both the surgeon performing MI induction and the investigators responsible for imaging processing, histological analysis, and molecular biology experiments. The animal experimental protocol (Fig. 1) was approved by the Institutional Animal Care and Use Committee of the First Hospital of Shanxi Medical University (Approval No. DWLL-2024-012). All procedures were conducted in accordance with the animal ethical principles and standards outlined in the Guide for the Care and Use of Laboratory Animals published by the US National Institutes of Health (NIH, 8th Edition, National Academies Press, 2011) and complied with the ARRIVE guidelines.

**Fig. 1.**
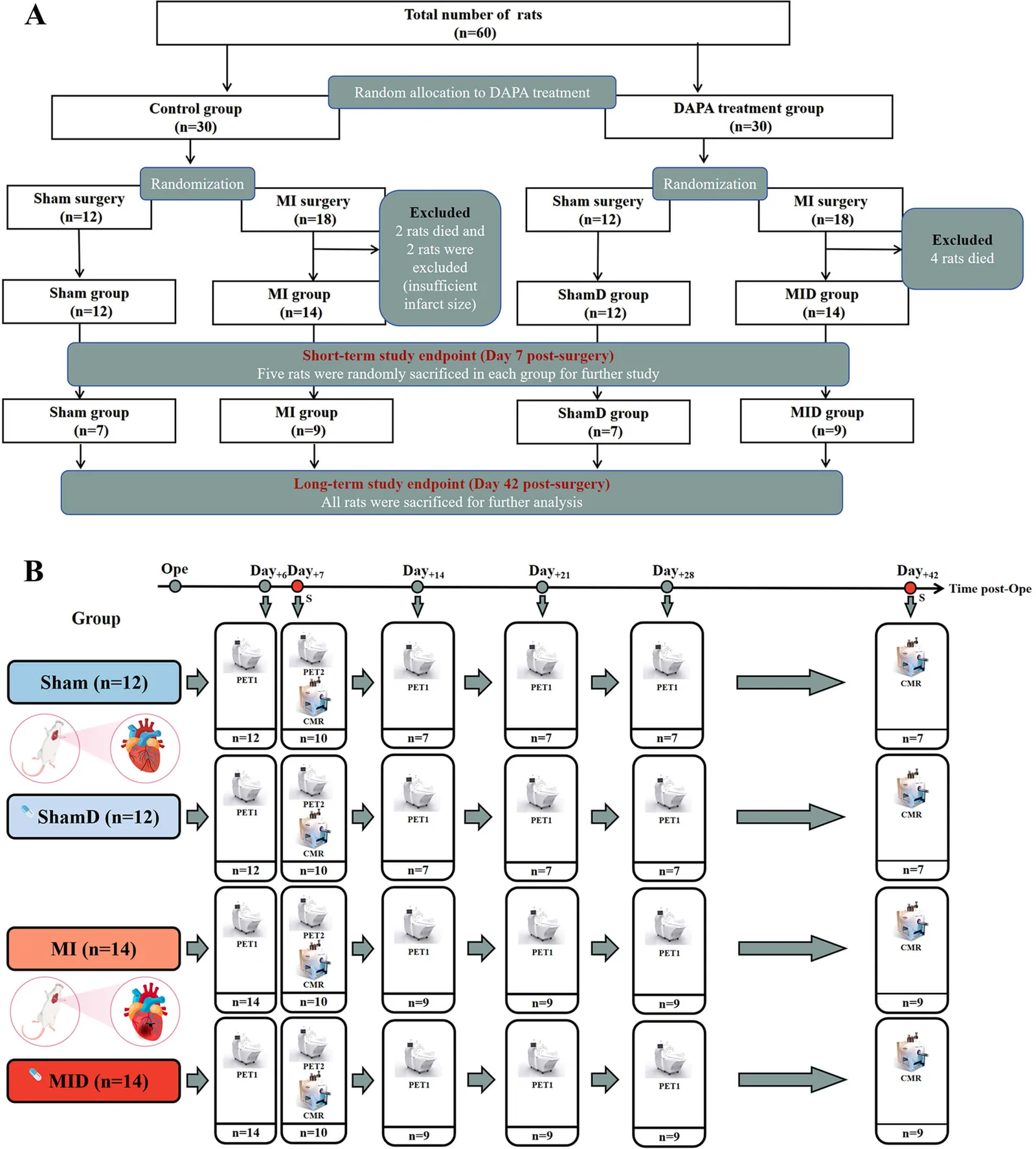
**Schematic overview of the *****in vivo***** experiments**. (A) Flowchart of animal allocation and grouping. (B) Timeline of longitudinal *in vivo *imaging assessments. S, Sacrifice; positron emission tomography (PET)1: ^18^F-fibroblast activation protein inhibitor-74 PET; PET2: ^18^F-fluorodeoxyglucose PET. Abbreviations: PET, positron emission tomography.

### 2.3 Measurement of BP and FBG

BP was measured in a quiet and temperature-controlled environment. Trained rats were gently placed in the animal BP monitor and restrained. Prior to measurement, the tail was warmed in water at 32 ℃ for 10–15 minutes (min) to dilate the tail artery. An inflatable tail cuff was attached to the base of the rat’s tail and adjusted to an appropriate tightness. Measurements were performed three times, and the mean of stable readings was recorded. If substantial variation was observed, measurements were repeated after a rest. Blood from the tail vein was used for FBG measurements with rats fasted for at least 6 h.

### 2.4 CMR Images Acquisition and Derivation of Cardiac Function Parameters

CMR was performed at baseline and at days 7 and 42 post-surgery using a 7T small animal MRI scanner (BioSpec 70/20 USR, Bruker Corporation, Ettlingen, Germany). Animals were rapidly anesthetized with isoflurane (2%, delivered in oxygen) and anesthesia was maintained at 1.25–1.5% throughout the imaging procedure. Then the animals were placed in the prone position with their heads facing the scanner. Body temperature was maintained at 37 ± 0.5 °C using a heating pad. After heart localization, consecutive short-axis (SAX) cine images were acquired using an ECG-triggered fast low-angle shot (FLASH) sequence, covering the entire left ventricle from apex to base. Both ECG and respiratory signals were continuously monitored throughout the scanning process to acquire high-quality images. The FLASH sequence parameters were: echo time = two ms, repetition time = 48 ms, eight repetitions, flip angle = 18 °, eight frames per cardiac cycle, field of view (FOV) = 60 × 60 mm^2^, matrix size = 192 × 192 and slice thickness = 1.5 mm. Image post-processing and analysis were performed in Segment (v4.0 R12067; Medviso AB, Lund, Sweden). SAX cine images were analyzed by manually delineating the LV endocardial and epicardial borders at both end-diastolic and end-systolic phases [35] in accordance with previously published methods. LV end-diastolic volume (LVEDV, mm^3^), LV end-systolic volume (LVESV, mm^3^), LV ejection fraction (LVEF, %) and LV mass (mg) were then calculated. Papillary muscles were excluded from the endocardial contour [35,36].

### 2.5 Cardiac Radionuclide Imaging and Quantitative Analysis

All PET scans were performed using a dedicated preclinical PET/CT scanner (IRIS PET/CT, Inviscan SAS, Strasbourg, France) and adhered to the same protocol. ^18^F-FDG PET was performed at baseline and 7 days after surgery. ^18^F-FAPI-74 PET was performed at baseline and on postoperative days 6, 14, 21, and 28. Prior to^ 18^F-FDG PET scanning, fasting was required for at least 6 h. ^18^F-FAPI-74 PET imaging does not require any special preparation. The synthesis of ^18^F-FDG and ^18^F-FAPI-74 was conducted as previously described procedures [37]. Isoflurane was used for anesthesia, as described in the CMR, and physiological parameters (body temperature, HR, and respiration) were monitored during imaging. Following tail vein injection of ^18^F-FDG or ^18^F-FAPI-74 (7-8 MBq), the rats were allowed a 60-min uptake period in the awake state. Static PET acquisition lasting 10 min was performed with attenuation correction achieved using a low-dose CT scan (parameters: 80 kV tube voltage and 1 mA tube current). All PET images were reconstructed using the three-dimensional ordered-subsets expectation maximization Monte Carlo algorithm (eight subsets and eight iterations) with a matrix size of 101 × 101 × 120, voxel size of 0.85 mm, and FOV of 80 × 95 mm, and the corresponding PET/CT fusion images were generated accordingly.

Myocardial ^18^F-FAPI-74 uptake and LV infarct volume were quantified using the PMOD software (version 4.1, PMOD Technologies LLC, Zurich, Switzerland). ^18^F-FAPI-74 accumulation was evaluated by drawing regions of interest (ROIs) on reconstructed PET/CT images and calculating the maximum standardized uptake value (SUV_max_) of the left ventricle [36]. The target-to-background ratio was calculated by dividing myocardial SUV_max_ by the liver mean standardized uptake value (SUV_mean_) (H/L ratio) to normalize background differences between rats. The infarcted myocardial volume of interest (VOI) was automatically generated using end-diastolic-gated ^18^F-FDG PET. Regions with an FDG uptake SUV_mean _<50% of that of the normal myocardium were defined as metabolic defects, with manual adjustments for accurate boundary delineation. The ratio of infarct VOI to LV volume was calculated to evaluate the infarct extent.

### 2.6 Histological Analysis

At study termination, excised hearts (sham-operated rats, n = 2 per group; MI rats, n = 4 per group) were preserved in 10% formalin (Cat No. G2160, Solarbio, Beijing, China) for 48 h. The specimens were then paraffin-embedded, sectioned at 4 μm and stained with Masson’s trichrome (MS) according to the manufacturer’s instructions. MS staining was applied to evaluate the extent of myocardial fibrosis. In infarcted hearts (heart tissue from the maximum short-axis plane collected 7 days after MI, n = 5), activated CFs were detected by FAP immunohistochemistry (IHC) staining. After deparaffinization, antigen retrieval, and blocking with 5% bovine serum albumin, cardiac sections were incubated with polyclonal rabbit anti-FAP antibodies (1:1000, Cat No. PA5-99313, Thermo Fisher Scientific, Waltham, MA, USA) overnight at 4 °C. After three washes, sections were exposed to secondary antibodies for 1 h at room temperature. All digital image analysis was performed with ImageJ software (NIH, USA).

CMR, PET, and digital histological images were independently analyzed by two experienced investigators following standardized processing protocols.

### 2.7 Real-Time Quantitative Polymerase Chain Reaction (RT-qPCR)

Myocardial tissue fragments were excised from the infarct border zone of infarcted hearts and from sham-operated hearts. Total RNA was extracted from myocardial tissues using the TRIzol Reagent Kit (Cat No. 93289, Sigma-Aldrich, St. Louis, MO, USA) according to the manufacturer’s instructions. After purification, total RNA was reverse-transcribed to generate complementary DNA using the High Capacity RNA-to-cDNA™ Kit (Cat No. 4387406, Thermo Fisher Scientific, Waltham, MA, USA). For RT-qPCR, amplification was carried out using the Power SYBR Green PCR Master Mix (Cat No. A46109, Thermo Fisher Scientific, Waltham, MA, USA) to evaluate the mRNA levels of markers associated with cardiac fibrosis and inflammation. Relative mRNA levels of target genes were quantified by normalization to *Actb *and determined by the 2^−ΔΔCT ^method. Table 1 summarizes the forward and reverse primer sequences used for amplification.

**Table 1.** **Primer sequences for real-time quantitative polymerase chain reaction analysis**.

Gene name	Forward Primer 5′——3′	Reverse Primer 5′——3′
*Col1a1*	ATGGTGAGACGTGGAAACCTG	CCTCCCGTTTTTGGGGGTT
*Col3a1*	CGGGCAAGAATGGAGCAAAG	GTGACCCATCTTTGCCGTCT
*Acta2*	TGACCCAGATTATGTTTGAGACCTT	AGAGTCCAGCACAATACCAGTT
*Nlrp3*	CCACAACTCACCCAAGGAGG	ACAGGCAACATGAGGGTCTG
*Pycard*	CAGCACAGGCAAGCACTCAT	CTGGTGGTCTCTGCACGAAC
*Casp1*	GACCGAGTGGTTCCCTCAAG	GACGTGTACGAGTGGGTGTT
*Il1b*	GAGTCTGCACAGTTCCCCAA	ATGTCCCGACCATTGCTGTT
*Il18*	AACAGCCAACGAATCCCAGA	GATAGGGTCACAGCCAGTCC
*Rela*	CCTGTTCCAAAGAGCACCCA	TGCCAGGTCTGTGAACACTC
*Actb*	TCTATGAGGGTTACGCGCTCC	CAGCTGTGGTGGTGAAGCTG

### 2.8 Western Blot (WB) Analysis

Frozen myocardial tissue from the infarct border zone was lysed in a protein lysis buffer supplemented with protease and phosphatase inhibitors to extract total protein. Protein concentrations were determined using the Pierce BCA Protein Assay Kit (Cat No. 23227, Abcam, Cambridge, UK). Equal amounts of protein lysates were separated by SDS-PAGE and electrotransferred onto PVDF membranes. The membranes were then blocked with 5% non-fat milk for 1 h at room temperature and incubated with primary antibodies overnight at 4 °C. On the following day, the membranes were subsequently incubated with appropriate secondary antibodies for 1 h at room temperature and visualized using an ECL detection system. Protein expression levels were normalized to β-actin. The primary antibodies used in this study included Collagen I (1:1000, Cat No. ab270993, Abcam, Cambridge, UK), Collagen III (1:1000, Cat No. ab184993, Abcam, Cambridge, UK), α-smooth muscle actin (α-SMA; 1:5000, Cat No. ab124964, Abcam, Cambridge, UK), NLRP3 (1:1000, Cat No. ab263899, Abcam, Cambridge, UK), apoptosis-associated speck-like protein containing a CARD (ASC; 1:500, Cat No. SAB4501315, Sigma-Aldrich, St. Louis, MO, USA), cleaved Casp-1 (1:1000, Cat No. PA5-99390, Thermo Fisher Scientific, Waltham, MA, USA), cleaved IL-1β (1:1000, Cat No. ab283818, Abcam, Cambridge, UK), cleaved IL-18 (1:5000, Cat No. ab191860, Abcam, Cambridge, UK), IκB (1:1000, Cat No. #9242, Cell Signaling Technology, Danvers, MA, USA), phospho-IκB (p-IκB, 1:1000, Cat No. #9246, Cell Signaling Technology, Danvers, MA, USA), nuclear factor kappa B (NF-κB, p65) (1:1000, Cat No. #8242, Cell Signaling Technology, Danvers, MA, USA), phospho-NF-κB (p-NF-κB, 1:1000, Cat No. #3033, Cell Signaling Technology, Danvers, MA, USA), FAP (1:1000, Cat No. PA5-99313, Thermo Fisher Scientific, Waltham, MA, USA), transforming growth factor beta 1 (TGF-β1; 1:1000, Cat No. MA5-16949, Thermo Fisher Scientific, Waltham, MA, USA), and β-actin (1:5000, Cat No. ab179467, Abcam, Cambridge, UK). For RT-qPCR and WB analyses, each group included five biological samples, with experiments repeated three times.

### 2.9 CF *In Vitro* Experiments

Commercially available rat CFs (Cat No. CP-R074, Procell, Wuhan, China) were derived from LV myocardial tissues of SD rats, characterized by immunofluorescence staining for Vimentin and α-SMA, and confirmed to be free of HIV-1, HBV, HCV, mycoplasma, bacteria, yeast, and fungi. The inflammatory CF model was established by stimulation with LPS, a specific ligand of Toll-like receptor 4 (TLR4), which induces *Nlrp3* mRNA expression in CFs via Toll-like receptor signaling in an NF-κB-dependent manner [8]. The cells were cultured in rat CF complete medium (Cat No. CM-R074, Procell, Wuhan, China) and, when they reached approximately 80% confluence, were digested with 0.25% trypsin (Cat No. PB180225, Procell, Wuhan, China). After centrifugation, the cell pellet was collected and resuspended. The dispersed cells were seeded into six-well plates (1 × 10^5^ cells/mL), and washed with pre-warmed phosphate-buffered saline (PBS; Cat No. PB180327, Procell, Wuhan, China) after 2 h. The medium was refreshed, followed by incubation for 6 h. Dose-response experiments were carried out to evaluate CF viability at different doses of LPS (0.01, 0.1, 1, 10, 100, 1000 ng/mL), DAPA (0.5, 1, 5, 10, 25, 50 μM), BAY 11-7082 (0.5, 1, 5, 10, 20, 30 μM) and MCC950 (0.01, 0.1, 1, 10, 100 μM), thereby determining the appropriate intervention concentrations. Cell viability and proliferation were measured using the CCK-8 reagent (Cat No. 96992-100TESTS-F, Sigma-Aldrich, St. Louis, MO, USA) following the standard procedures provided by the manufacturer.

Based on the cell viability results obtained from the concentration gradient experiments, the cells were stimulated with 10 ng/mL LPS (Cat No. 00-4976-93, Thermo Fisher Scientific, Waltham, MA, USA) for 12 h. The control group was administered PBS alone without LPS stimulation. For DAPA treatment, the cells were pre-incubated with DAPA (6, 8, or 10 μM) for 30 min before LPS stimulation. In addition, to clarify whether DAPA attenuates NLRP3 inflammasome activation in a NF-κB-dependent manner or through direct suppression of NLRP3, two parallel blockade groups were established: DAPA (10 μM) plus BAY 11-7082 (12 μM) (a specific NF-κB inhibitor; Cat No. HY-13453, CAS: 19542-67-7, MedChemExpress, Monmouth Junction, NJ, USA) and DAPA (10 μM) plus MCC950 (10 μM) (a selective NLRP3 inflammasome inhibitor; Cat No. HY-12815, CAS: 210826-40-7, MedChemExpress, Monmouth Junction, NJ, USA). These groups were treated following the same procedures as the DAPA intervention alone. Experiments were performed in six technical replicates and independently repeated five times. Subsequent biological assays were performed. Protein expressions were normalized to β-actin, and the mean values of technical replicates from five independent experiments were used for statistical analysis.

### 2.10 Statistical Analysis

GraphPad Prism software (version 10.1.2, GraphPad, Boston, MA, USA) was employed for statistical analysis. All data were analyzed using parametric statistical tests under an a priori assumption of normality and are expressed as means ± standard deviations (SDs). Mean differences (mean diff.) with 95% confidence intervals (CIs) were calculated for all comparisons and reported as the experimental group (MI or MID group) - the sham-operated group (Sham or ShamD group), and as MID group - MI group. Two-group comparisons were conducted using an unpaired two-tailed Student’s *t*-test. Comparisons across three or more groups were performed using one-way analysis of variance (ANOVA) with Tukey’s multiple comparison test. Repeated measurements were analyzed using a mixed-effects model with time and treatment as fixed effects and a random intercept for each rat. Interaction between group and time point was included to assess differences in the time course, and Tukey’s post hoc test was used for pairwise comparisons if significant. Differences were considered statistically significant when the *p*-value was <0.05.

## 3. Results

### 3.1 Physiological Characteristics in Non-Diabetic MI Rats After DAPA Treatment

As illustrated in Fig. 2, baseline physiological parameters showed no significant intergroup differences prior to surgery (all *p *> 0.05). Longitudinal mixed-effects analysis demonstrated that BW increased significantly over time across all groups (F _(2.466, 69.05) _= 3474. 41, *p *< 0.0001), with no significant time × group interaction (F _(9, 84)_ = 0.4064, *p *= 0.928) (Fig. 2A). FBG and HR remained stable, with no significant group differences or time effects (Fig. 2B,C). These findings indicate that DAPA does not significantly affect BW, glycemic status, or HR in non-diabetic rats.

**Fig. 2.**
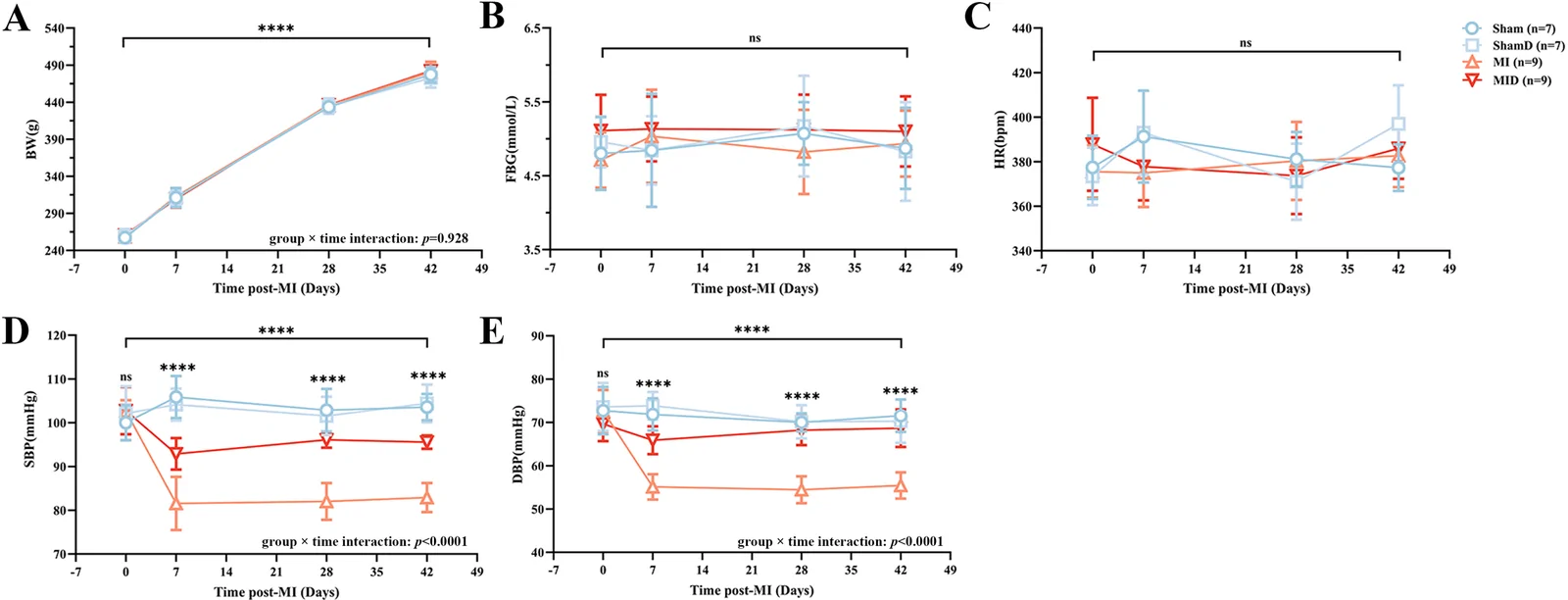
**Physiological parameters were measured at baseline and on days 7, 28, and 42 after myocardial infarction (MI)**. (A–C), Dapagliflozin had no significant impact on body weight (A), fasting blood glucose (B), or heart rate (C) in non-diabetic MI rats. (D,E) Dapagliflozin significantly improved both systolic blood pressure (D) and diastolic blood pressure post-MI (E). Sham and ShamD groups (n = 7); MI and MID groups (n = 9). Data are presented as means ± standard deviations (SDs). Longitudinal comparisons: mixed-effects model with Tukey’s test. Statistical significance: *p *< 0.05. ns: no significant difference. **** denotes statistical significance: *p *< 0.0001. Abbreviations: MI, myocardial infarction.

As shown in Fig. 2D and E, systolic BP (SBP) and diastolic BP (DBP) differed significantly across groups over time (SBP: F _(2.565, 71.82) _= 16. 45, *p *< 0.0001; DBP: F _(2.735, 76.57) _= 20. 27, *p *< 0.0001), with a significant time × group interaction (SBP: F_ (9, 84)_ = 16.09, *p *< 0.0001; DBP: F_ (9, 84)_ = 10.66, *p *< 0.0001). At day 7 post-MI, compared with sham-operated rats, MI rats showed a marked decrease in both SBP and DBP (all *p *< 0.0001), reflecting impaired cardiac function. In the MID group, SBP and DBP remained lower than those in the sham group, but were significantly higher than those in the MI group (SBP: vs. MI group, mean diff.: 11.33 mmHg, 95% CI: 4.41 to 18.26 mmHg, *p* = 0.0017; DBP: vs. MI group, mean diff.: 10.78 mmHg, 95% CI: 6.62 to 14.94 mmHg, *p *< 0.0001). Similarly, on days 28 and 42 post-MI, DAPA-treated MI rats exhibited improved SBP and DBP compared with untreated MI rats (all *p *< 0.05). Notably, DAPA did not affect SBP or DBP in sham-operated rats (all *p *> 0.05). No deaths occurred in any of the groups during the observation period.

### 3.2 DAPA Ameliorated LV Performance After MI as Assessed by CMR

Twenty randomly selected rats were subjected to baseline *in vivo* imaging assessments. All rats exhibited normal myocardial viability, CF activity, and cardiac morphology and function (detailed information is provided in **Supplementary Table 2**). Given the close association between initial infarct extent and cardiac function, as well as subsequent myocardial fibrosis and ventricular remodeling, baseline infarct size was quantified by ^18^F-FDG PET on day 7 post-infarction. No significant differences were observed in infarct volume fraction between the MI and MID groups (**Supplementary Fig. 1**), indicating comparable severity of myocardial injury.

Fig. 3 shows the LV performance assessed by CMR on days 7 and 42 after MI surgery. At day 7 post-MI (Fig. 3A), the average LVESV was significantly higher in the MI group (135.7 ± 7.5 mm^3^) than in the Sham (97.7 ± 4.4 mm^3^, mean diff.: 38.04 mm^3^, 95% CI: 30.62 to 45.46 mm^3^, *p *< 0.0001) and ShamD (99.4 ± 4.1 mm^3^, mean diff.: 36.33 mm^3^, 95% CI: 28.91 to 43.75 mm^3^, *p *< 0.0001) groups. Correspondingly, the average LVEF of MI rats was significantly decreased (MI group 56.0 ± 2.5% vs. Sham group 67.6 ± 1.4%, mean diff.: –11.60%, 95% CI: –14.16 to –9.04%, *p *< 0.0001; vs. ShamD group 66.9 ± 1.7%, mean diff.: –10.90%, 95% CI: –13.46 to –8.34%, *p *< 0.0001). MI rats receiving DAPA exhibited lower LVESV (119.8 ± 7.0 mm^3^ vs. MI group, mean diff.: –15.97 mm^3^, 95% CI: –23.39 to –8.55 mm^3^, *p *< 0.0001) and higher LVEF (60.8 ± 2.7% vs. MI group, mean diff.: 4.80%, 95% CI: 2.24 to 7.36%, *p *< 0.0001), than untreated MI rats. There were no statistically significant differences in the LVEDV or LV mass among the four groups (all *p *> 0.05).

**Fig. 3.**
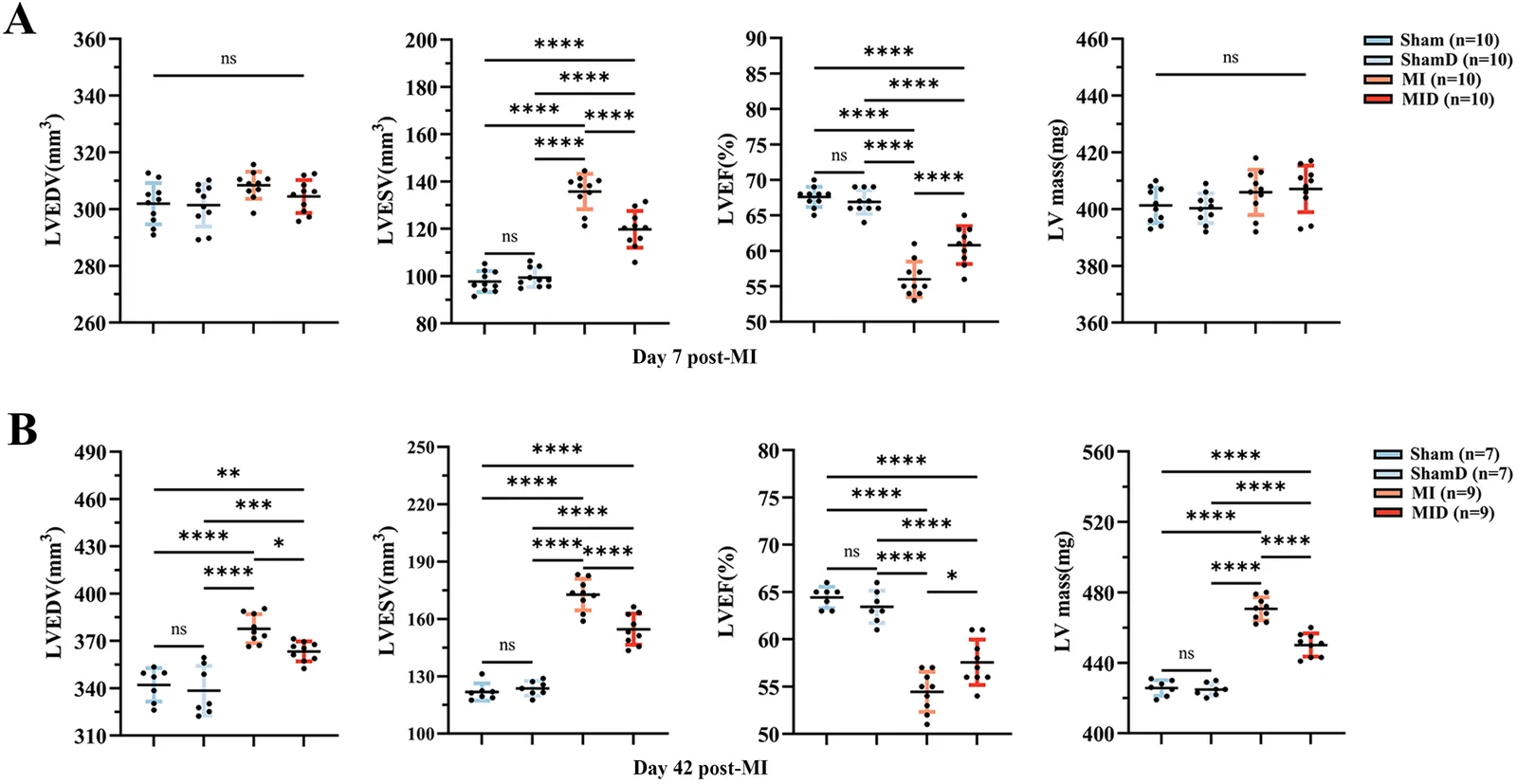
**Left ventricular (LV) performance assessed by cardiac magnetic resonance on days 7 and 42 after myocardial infarction (MI)**. (A) Dapagliflozin improved early LV systolic function post-MI (n = 10 per group). (B) Long-term dapagliflozin treatment preserved LV systolic function and mitigated adverse cardiac remodeling following MI (Sham and ShamD groups: n = 7; MI and MID groups: n = 9). Data are presented as means ± SDs. Comparisons among three or more groups: one-way ANOVA with Tukey’s test. Statistical significance: *p *< 0.05. ns: no significant difference. *, **, ***, and **** denote statistical significance at *p *< 0.05, *p *< 0.01, *p *< 0.001, and *p *< 0.0001, respectively. Abbreviations: LV, left ventricle; MI, myocardial infarction.

Subsequent observations revealed that MI rats experienced progressive ventricular dilation (increased LVEDV and LVESV), reflecting ongoing LV remodeling. CMR assessment at the study endpoint (day 42 post-MI) (Fig. 3B) demonstrated that the MI hearts exhibited a marked decrease in LVEF (54.4 ± 2.1% vs. Sham group, mean diff.: –9.98%, 95% CI: –12.68 to –7.28%, *p *< 0.0001), along with increases in LVEDV (377.7 ± 9.2 mm^3^ vs. Sham group, mean diff.: 35.60 mm^3^, 95% CI: 21.02 to 50.18 mm^3^), LVESV (172.7 ± 8.2 mm^3^ vs. Sham group, mean diff.: 50.93 mm^3^, 95% CI: 41.60 to 60.27 mm^3^), and LV mass (470.7 ± 6.5 mg vs. Sham group, mean diff.: 44.95 mg, 95% CI: 37.22 to 52.68 mg) (all *p *< 0.0001). Long-term treatment with DAPA significantly improved these parameters. LVEF was preserved (57.6 ± 2.4% vs. MI group, mean diff.: 3.11%, 95% CI: 0.58 to 5.64%, *p *= 0.011). The LVEDV (363.3 ± 6.3 mm^3^ vs. MI group, mean diff.: –14.39 mm^3^, 95% CI: –28.03 to –0.75 mm^3^, *p *= 0.036), LVESV (154.6 ± 8.2 mm^3^ vs. MI group, mean diff.: –18.11 mm^3^, 95% CI: –26.84 to –9.38 mm^3^, *p *< 0.0001), and LV mass (450.1 ± 6.6 mg vs. MI group, mean diff.: –20.56 mg, 95% CI: –27.78 to –13.33 mg,* p *< 0.0001) were significantly reduced compared with untreated MI rats.

### 3.3 DAPA Attenuated Post-MI LV Fibrosis

At 42 days post-MI, MS staining revealed pronounced LV wall thinning and an extensive area of blue-stained fibrosis in untreated MI rats. In contrast, the extent of myocardial fibrosis was markedly ameliorated in the MID group rats (20.1 ± 1.9% vs. MI group 31.1 ± 4.0%), with a mean diff. of –10.95% (95% CI, –16.38 to –5.52%; *p *= 0.0026) (Fig. 4A,B). Consistently, the mRNA levels of fibrotic markers (*Col1a1*, *Col3a1*, and* Acta2*) were significantly up-regulated in myocardial tissue from MI rats. Their corresponding proteins (Collagen I, Collagen III, and α-SMA) expression was also significantly increased, but were markedly reduced following DAPA administration (all* p *< 0.05) (Fig. 4C,D).

**Fig. 4.**
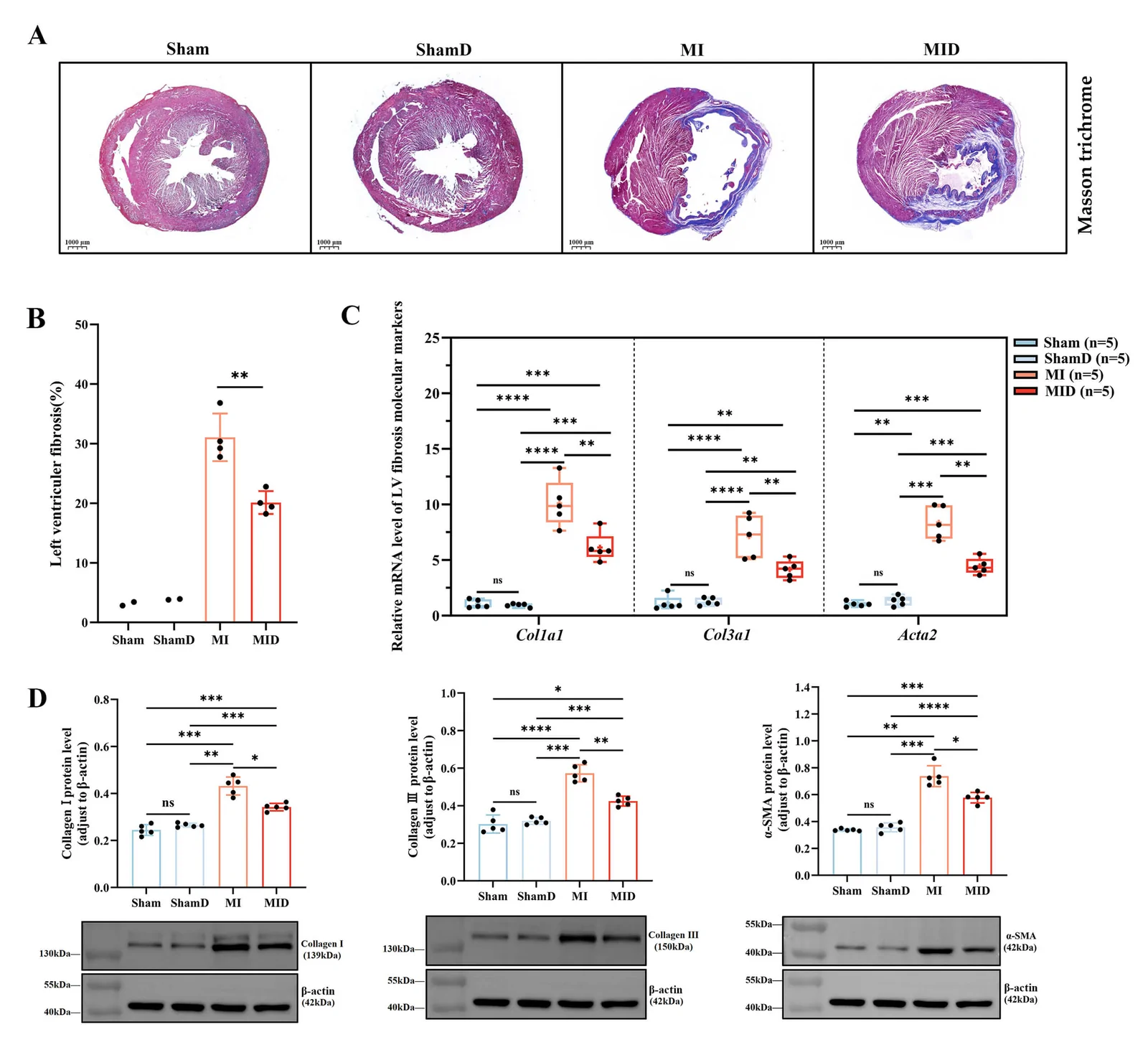
**Evaluation of left ventricular (LV) fibrosis at Day 42 after myocardial infarction (MI)**. (A,B) Cardiac Masson’s trichrome staining revealed reduced LV fibrosis after dapagliflozin treatment (Sham and ShamD groups: n = 2, displayed for reference, not included in comparisons; MI and MID groups: n = 4). Scale bar = 1000 μm; original magnification: 1.5 × objective. (C,D) Real-time quantitative polymerase chain reaction (C) and western blot analysis (D) showed reduced expression of fibrosis molecular markers in the MID group rats (n = 5 per group). Data are presented as means ± SDs. Two-group comparisons: unpaired two-tailed Student’s *t*-test; comparisons among three or more groups: one-way ANOVA with Tukey’s test. The displayed blots were cropped for clarity and uncropped original blots are presented in **Supplementary Fig. 2**. Statistical significance: *p *< 0.05. ns: no significant difference. *, **, ***, and **** denote statistical significance at* p *< 0.05, *p *< 0.01, *p *< 0.001, and *p *< 0.0001, respectively. Abbreviations: LV, left ventricle; MI, myocardial infarction.

### 3.4 DAPA Inhibited CF Activation in Infarcted Hearts as Evaluated by ^18^F-FAPI-74 PET

Representative ^18^F-FDG and ^18^F-FAPI-74 PET images obtained one week postoperatively are presented in Fig. 5A. ^18^F-FDG PET showed normal and homogeneous myocardial uptake in both normal and sham-operated rats. However, MI hearts exhibited focal defects in ^18^F-FDG uptake, corresponding to the infarcted region where marked ^18^F-FAPI-74 accumulation was observed.

**Fig. 5.**
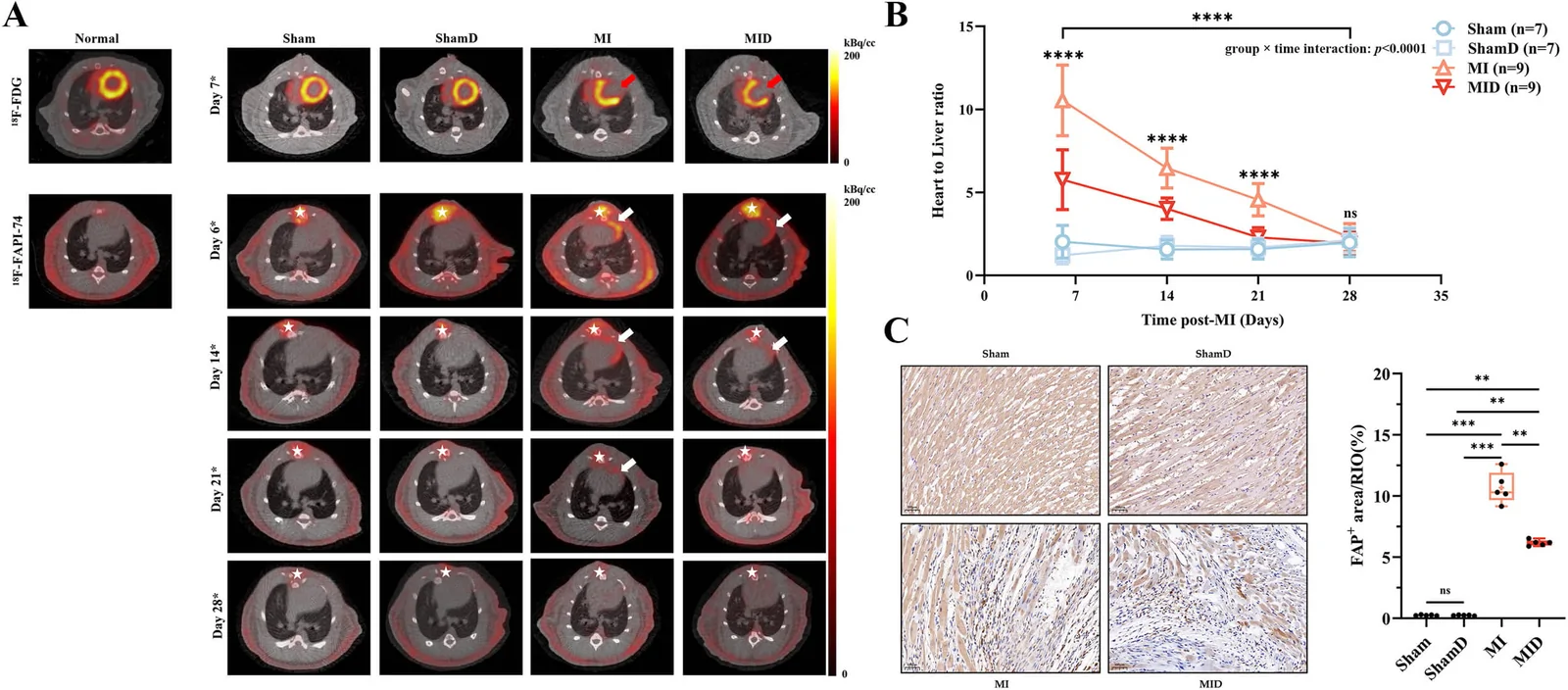
**Longitudinal *****in vivo***** assessment of cardiac fibroblast (CF) activation on days 6, 14, 21 and 28 after myocardial infarction (MI)**. (A) Representative myocardial ^18^F-fibroblast activation protein inhibitor-74 (^18^F-FAPI-74) positron emission tomography images. The red arrow indicates an ^18^F-fluorodeoxyglucose uptake defect in the infarcted myocardium. The white arrow indicates the ^18^F-FAPI-74 uptake in the infarcted myocardium and the white star indicates the tracer accumulation at the surgical incision site. (B) Dapagliflozin inhibited CF activation post-MI, as evidenced by reduced myocardial ^18^F-FAPI-74 uptake in the infarct region (Sham and ShamD groups: n = 7; MI and MID groups: n = 9). (C) Fibroblast activation protein expression was validated by cardiac immunohistochemistry staining (n = 5 per group). Scale bar = 100 μm; original magnification: 20 × objective. *: post-surgery. Data are presented as means ± SDs. Longitudinal comparisons: mixed-effects model with Tukey’s test; comparisons among three or more groups: one-way ANOVA with Tukey’s test. Statistical significance: *p *< 0.05. ns: no significant difference. *, **, ***, and **** denote statistical significance at *p *< 0.05, *p *< 0.01, *p *< 0.001, and *p *< 0.0001, respectively. Abbreviations: CF, cardiac fibroblast; MI, myocardial infarction; ^18^F-FAPI-74, ^18^F-fibroblast activation protein inhibitor-74; DAPA, dapagliflozin.

Longitudinal mixed-effects analysis revealed a significant time effect (F_ (1.920, 53.76)_ = 45.13, *p *< 0.0001) and a significant time ×group interaction (F_ (9, 84) _= 23.49, *p *< 0.0001) in myocardial^ 18^F-FAPI-74 uptake. As can be seen from Fig. 5B, serial ^18^F-FAPI-74 PET imaging indicated that myocardial ^18^F-FAPI-74 uptake peaked on day 6 post-MI (H/L ratio, 10.78 ± 2.14) and declined over time, returning to baseline levels by day 28 (H/L ratio, 6.46 ± 1.20 at Day 14 post-MI; 4.56 ± 0.97 at Day 21 post-MI; 2.28 ± 0.84 at Day 28 post-MI). DAPA reduced it in the infarcted hearts. DAPA-treated MI rats had significantly lower peak myocardial uptake than untreated MI rats at day 6 post-MI (H/L ratio: MID group 5.68 ± 1.73 vs. MI group, mean diff.: –4.77, 95% CI: –7.44 to –2.10, *p *= 0.0006). At day 14, the MI group exhibited the highest myocardial uptake (H/L ratio: MID group 4.01 ± 0.65 vs. MI group, mean diff.: –2.45, 95% CI: –3.79 to –1.11, *p *= 0.0007). At day 21, no obvious myocardial uptake was observed in MID group (H/L ratio: MID group 2.29 ± 0.59 vs. Sham group 1.58 ± 0.60, *p* = 0.135; vs. ShamD group 1.68 ± 0.58, *p* = 0.214), however, the MI group still exhibited detectable myocardial uptake (H/L ratio: vs. MID group, mean diff.: –2.27, 95% CI: –3.38 to –1.16, *p *= 0.0002). FAP-IHC of cardiac sections performed 7 days after MI revealed marked infiltration of FAP-positive cells in the infarct border zone, which was not observed in the normal myocardium. This infiltration was significantly attenuated by DAPA treatment (Fig. 5C).

### 3.5 DAPA Suppresses NLRP3-Dependent Inflammatory Signaling in Infarcted Cardiac Tissue

To investigate the early anti-inflammatory effects of DAPA on NLRP3-dependent signaling, the ventricular expression levels of NLRP3 inflammasome components (NLRP3, ASC, and Casp-1) and the associated cytokines (IL-1β and IL-18) were assessed in sham-operated hearts and in border-zone tissues from infarcted hearts collected at day 7 post-surgery. The mRNA levels of *Nlrp3, Pycard, Casp1, Il1b, and Il18* were low in non-ischemic hearts, whereas they were significantly elevated in infarcted hearts. Similarly, the corresponding proteins expression was significantly increased in infarcted hearts. (all *p *< 0.05) (Fig. 6A,B). As expected, in the MID group, this overexpression was attenuated and the expression levels of the above inflammatory markers were significantly reduced (all *p *< 0.05).

**Fig. 6.**
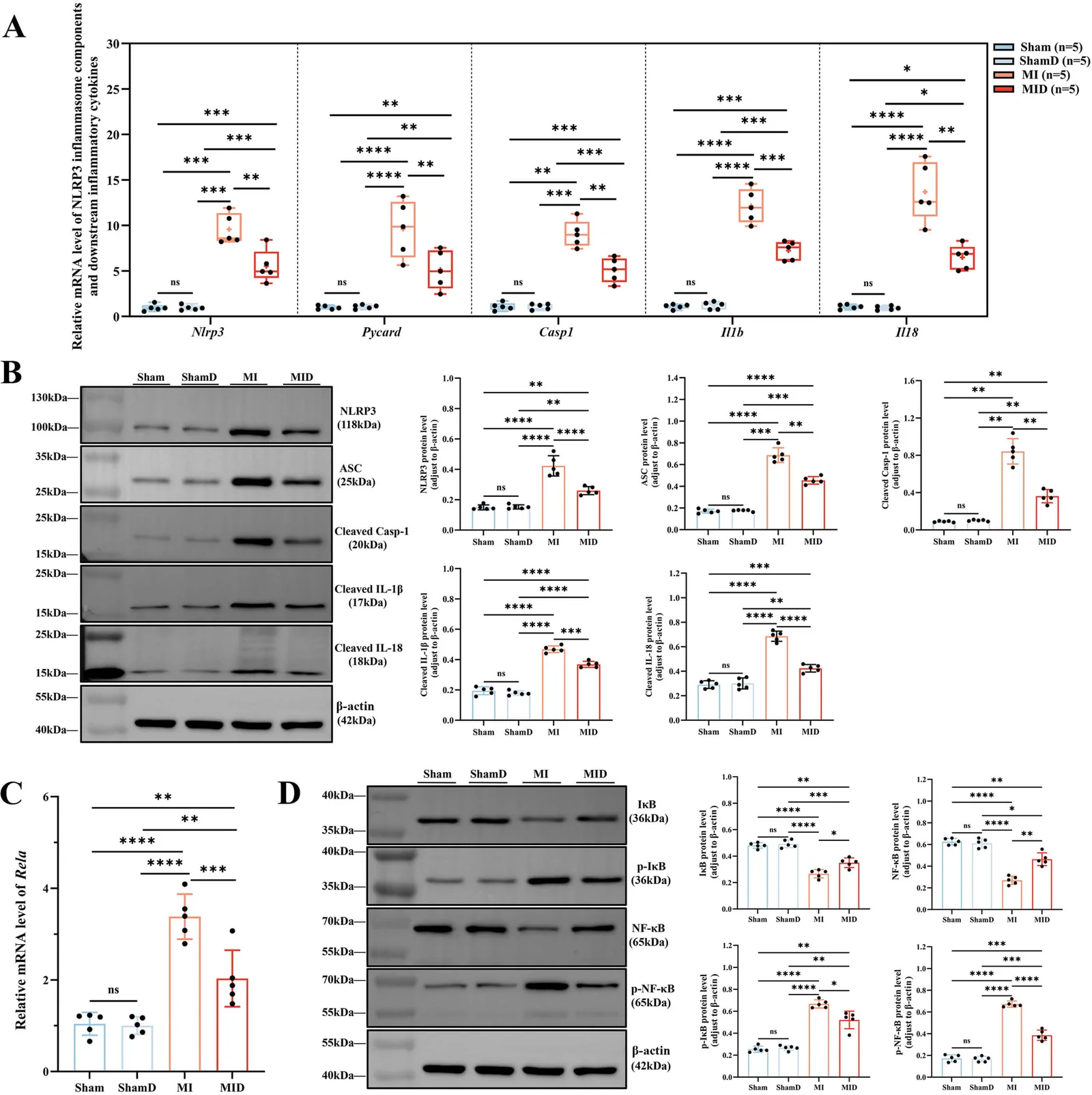
**Effects of dapagliflozin (DAPA) on the NACHT, LRR, and PYD domains-containing protein 3 (NLRP3)-dependent signaling in infarcted cardiac tissue**. (A,B) Real-time quantitative polymerase chain reaction (RT-qPCR) (A) and western blot (WB) (B) analyses demonstrated that DAPA attenuated NLRP3-dependent inflammation in infarcted cardiac tissue. (C,D) RT-qPCR (C) and WB (D) analyses demonstrated that DAPA suppressed nuclear factor kappa B pathway activation. The displayed blots were cropped for clarity and uncropped original blots are presented in **Supplementary Fig. 2**. n = 5 per group. Data are presented as means ± SDs. Comparisons among three or more groups: one-way ANOVA with Tukey’s test. Statistical significance: *p *< 0.05. ns: no significant difference. *, **, ***, and **** denote statistical significance at* p *< 0.05, *p *< 0.01, *p *< 0.001, and *p *< 0.0001, respectively. Abbreviations: DAPA, dapagliflozin; NLRP3, NACHT, LRR, and PYD domains-containing protein 3; RT-qPCR, real-time quantitative polymerase chain reaction; WB, western blot.

Accordingly, NF-κB, an upstream regulator of the NLRP3 inflammasome activation, was also evaluated. NF-κB pathway activation was evidenced by the up-regulation of *Rela *mRNA (Fig. 6C), alongside increased phosphorylation and concomitant decreased expression of both of IκB and NF-κB in infarcted hearts (all *p *< 0.05) (Fig. 6D). DAPA markedly suppressed NF-κB pathway activation.

### 3.6 DAPA Suppresses NF-κB/NLRP3 Pathway and Phenotypic Transition in LPS-Stimulated CFs

We assessed the effects of different concentrations of LPS, DAPA, BAY 11-7082, and MCC950 on CF viability. According to the results presented in Fig. 7A, LPS concentrations below 10 ng/mL did not affect cell viability, whereas concentrations of 10 and 100 ng/mL significantly increased cell viability. In contrast, 1000 ng/mL led to a significant reduction in cell viability. BAY 11-7082 at concentrations of 0.5 to 20 μM had no significant impact on CF viability, while 30 μM markedly reduced cell viability. MCC950 exhibited minimal cytotoxicity within the range of 0.01–10 μM, whereas a high concentration of 100 μM significantly suppressed CF viability. Accordingly, based on these results, cells were pre-incubated with 6, 8, and 10 μM DAPA for 30 mins, followed by 12-h stimulation with 10 ng/mL LPS for subsequent *in vitro* experiments. BAY 11-7082 and MCC950 were used at concentrations of 12 μM and 10 μM, respectively, for pathway inhibition experiments. As illustrated in Fig. 7B, DAPA treatment significantly suppressed LPS-induced CF proliferation in a concentration-dependent manner. Both combination treatment groups exhibited greater inhibition of cell proliferation compared with DAPA treatment alone.

**Fig. 7.**
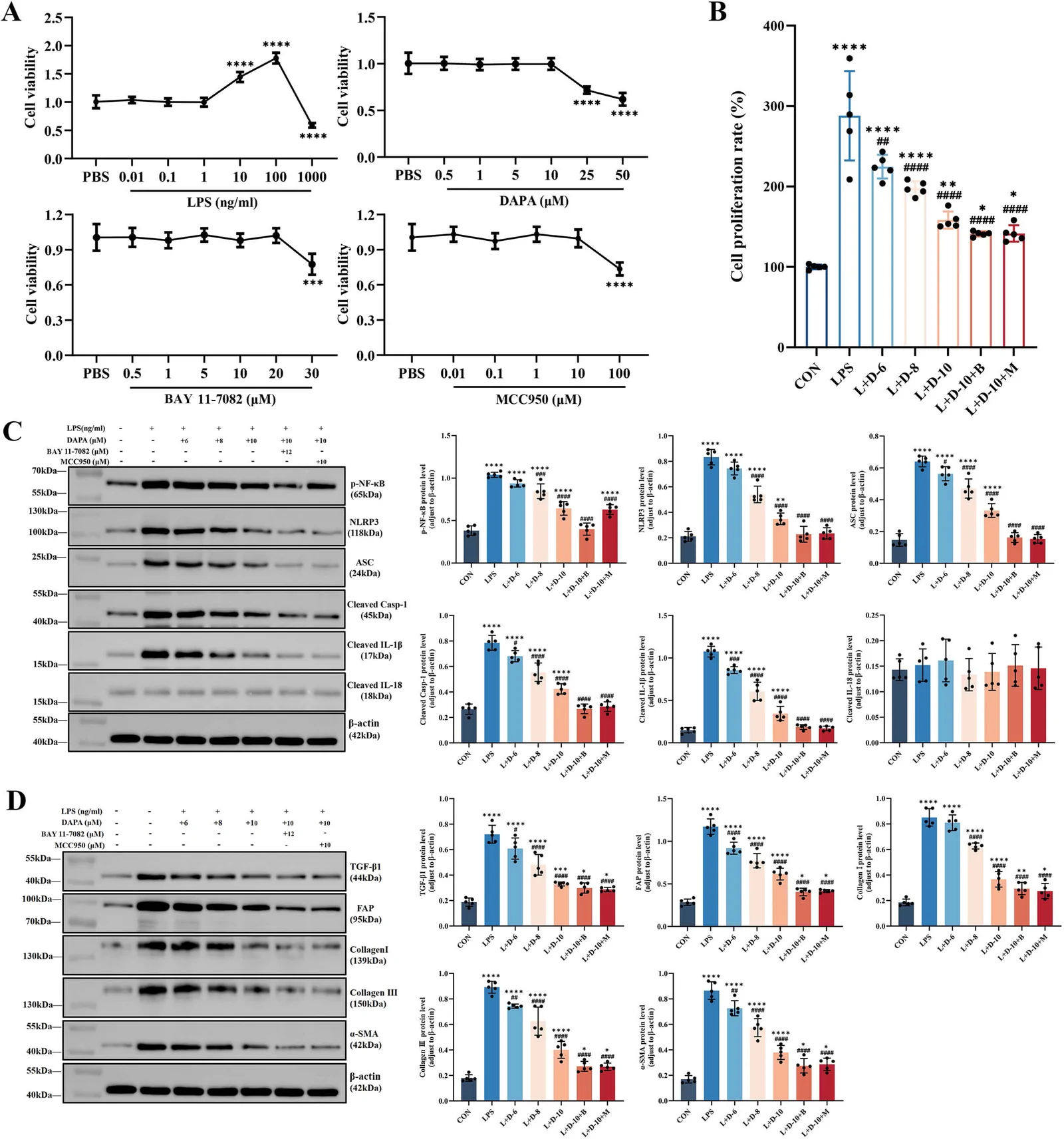
**Effects of dapagliflozin (DAPA) on lipopolysaccharide (LPS)-stimulated NACHT, LRR, and PYD domains-containing protein 3 (NLRP3) inflammasome activation and phenotypic transition in cardiac fibroblasts (CFs) (n = 5)**. (A) Dose-dependent effects of LPS, DAPA, BAY 11-7082, and MCC950 on the viability of CFs. (B) DAPA reduced LPS-stimulated proliferation of CFs. (C) DAPA attenuates nuclear factor kappa B/NLRP3 pathway activation in LPS-treated CFs. (D) DAPA inhibited phenotypic transition in LPS-treated CFs. The displayed blots were cropped for clarity and uncropped original blots are presented in **Supplementary Fig. 2**. n = 5 per group. Comparisons among three or more groups: one-way ANOVA with Tukey’s test. Data are presented as means ± SDs. Statistical significance: *p *< 0.05. *, **, ***, and **** denote statistical significance vs. CON (*p *< 0.05, 0.01, 0.001, and 0.0001, respectively); #, ##, ###, and #### denote statistical significance vs. LPS (*p *< 0.05, 0.01, 0.001, and 0.0001, respectively). Abbreviations: DAPA, dapagliflozin; LPS, lipopolysaccharide; NLRP3, NACHT, LRR, and PYD domains-containing protein 3; CFs, cardiac fibroblasts.

Fig. 7C showed that LPS triggered NF-κB phosphorylation and NLRP3 inflammasome activation in cultured CFs. This was accompanied by CF activation and enhanced collagen synthesis. WB analyses demonstrated that the expression of p-NF-κB, NLRP3, ASC, cleaved Casp-1 and cleaved IL-1β was significantly increased. No difference in IL-18 expression was observed between LPS-treated and untreated CFs. Consistently, markers associated with fibroblast phenotype (TGF-β1 and FAP) and collagen synthesis (CollagenⅠ, Collagen Ⅲ and α-SMA) showed marked upregulation (Fig. 7D). A 30-min DAPA pre-treatment significantly reduced the protein expression of NF-κB/NLRP3 pathway, along with markers of phenotypic transition and collagen synthesis. The inhibitory effects were dose dependent, with greater suppression observed at higher doses. Combined treatment with DAPA and BAY 11-7082 markedly suppressed the phosphorylation of NF-κB and activation of the NLRP3 inflammasome to levels comparable to those in the PBS group. In the group treated with DAPA and MCC950, the expression of NLRP3 inflammasome components and down-stream inflammatory mediators was significantly reduced to PBS group levels also, whereas p-NF-κB expression level remained comparable to that in the DAPA-treated alone group. Concurrently, the markers of fibroblast phenotypic transition and collagen synthesis were substantially decreased in the two combination treatment groups.

## 4. Discussion

Preclinical studies have indicated that targeting NLRP3-dependent signaling may interrupt the vicious cycle of post-MI inflammation and fibrotic remodeling. In this study, we observed that DAPA modulated NLRP3-dependent signaling in infarcted cardiac tissues and CFs. *In vitro* experiments demonstrated that DAPA regulated CF proliferation and phenotypic transition. These results further extend the understanding of its anti-fibrotic effects after MI and elucidate the potential molecular mechanisms. They also clarified the close relationship between NLRP3 inflammasome activation in CFs and their aberrant activation, highlighting the critical regulatory role of NLRP3-dependent signaling in the progression of post-MI myocardial fibrosis. In addition, ^18^F-FAPI-74 PET was used for the first time to visualize the inhibitory effect of the SGLT2is DAPA on CF activation* in vivo*. This study not only dynamically revealed the benefits of DAPA in post-infarction myocardial fibrosis but also emphasized the potential utility of FAP-targeted PET in evaluating the therapeutic efficacy of anti-fibrotic treatments.

In addition to its well-known glucose-lowering properties, the cardioprotective effects of SGLT2is have been attributed mainly to improved myocardial contractility through the attenuation of post-MI fibrosis. As previously reported, our results showed that DAPA attenuated myocardial fibrosis and ventricular dilation while improving cardiac function in male non-diabetic rats after MI; these findings further support the clinical utility of SGLT2is in non-diabetic patients [15,20,34]. Additionally, our* ex vivo* analysis demonstrated that DAPA significantly suppressed NLRP3 inflammasome activation in early-infarct hearts, further validating NLRP3 as a promising therapeutic target for attenuating adverse remodeling after MI. Moreover, a significant decrease in NF-κB phosphorylation was observed, suggesting that NF-κB inhibition contributes to the regulation of NLRP3-dependent inflammation. Notably, the safety and efficacy of post-MI anti-inflammatory therapies depend critically on patient stratification, timing of administration, agent selection, and regimen optimization [5]. Xu Chen et al. [38] reported that a transient increase in NLRP3 inflammasome expression was observed in the infarcted myocardium, peaking on day 3 post-MI and resolving by day 14. To allow effective modulation of NLRP3-dependent signaling at the onset of the early inflammatory surge post-infarction, DAPA was administered one week prior to MI induction. In addition, given the widespread first-line use of SGLT2is in patients with type 2 diabetes (T2D), a major risk factor for MI, pre-administration partially recapitulates the clinical scenario in which some T2D patients might already have been receiving these drugs before MI. However, this protocol does not capture the clinical scenario in which treatment begins after the onset of MI. Similarly, in the context of MI, the anti-inflammatory efficacy of SGLT2is may be influenced by the timing of administration, patient-specific factors (e.g., glycemic status, inflammatory burden, and sex), as well as dosing and treatment duration. Future studies should systematically investigate these variables to comprehensively elucidate their effects on NLRP3-dependent signaling and to identify optimized therapeutic strategies.

Resident CFs act as sentinel cells in cardiac tissue [39] that detect myocardial injury signals, are rapidly activated, and contribute to inflammatory responses and extracellular matrix remodeling after MI. The activation of CFs represents a pivotal molecular event in post-infarction myocardial fibrosis; furthermore, its the extent, duration, and spatiotemporal dynamics directly influence the progression of ventricular remodeling [40,41]. CF activation is a promising imaging target for the early detection and treatment of post-MI fibrosis [42,43]. In this study, serial dynamic ^18^F-FAPI-74 PET was performed to systematically evaluate the temporal changes in CF activation in MI rats after DAPA treatment on postoperative days 6, 14, 21, and 28. The first ^18^F-FAPI-74 PET scan was performed on day 6 post-MI, because myocardial uptake peaks at this time point [29]. Consistent with previous reports, these results demonstrated that MI rats showed marked myocardial ^18^F-FAPI-74 uptake in the infarcted region on day 6, followed by a gradual decline, and a return to baseline approximately 1 month after MI. DAPA treatment significantly reduced the peak uptake and promoted an earlier return to baseline by day 21. These results suggest that ^18^F-FAPI-74 PET enables noninvasive longitudinal tracking and quantitative assessment of CF activation following MI with DAPA treatment, and may serve as a valuable tool for monitoring the therapeutic efficacy of anti-fibrotic therapy. Beyond cardiovascular disease, molecular imaging has also shown promise for therapeutic monitoring and treatment guidance across various disease settings [44]. IHC analysis of FAP on day 7 verified that fewer FAP^+^ cells were present in the infarct border zone after DAPA treatment. Tillmanns et al. [31] provided evidence that FAP^+^ cells in the infarct border zone extensively co-express prolyl-4-hydroxylase β, α-SMA, and vimentin, supporting their identity as activated CFs. In contrast, other cell types, including pericytes, vascular smooth muscle cells, endothelial cells, and macrophages, exhibit only minimal FAP expression. Therefore, FAPI uptake in the infarcted hearts mainly originates from activated CFs. Together, these imaging and histological findings indicate that DAPA effectively suppressed CF activation after MI.

In* in vitro* experiments, CFs were exposed to LPS to mimic TLR4-NF‑κB pathway activation by endogenous damage-associated molecular patterns (DAMPs) within the infarcted myocardium microenvironment, as a proof-of-concept. Although LPS is a pathogen-associated molecular pattern, it induces NLRP3-dependent inflammation that closely mirrors the signaling cascade triggered by Toll-like receptors (TLRs) in response to DAMPs during MI [8]. *In vitro *results showed that DAPA attenuated NLRP3 inflammasome activation, the downstream inflammatory mediators and NF-κB phosphorylation in LPS-induced CFs. Furthermore, in the group co-treated with BAY 11-7082, the NF-κB/NLRP3 pathway in CFs was almost completely suppressed to control levels. MCC950 selectively attenuated NLRP3 inflammasome activation, whereas phosphorylated NF-κB levels remained comparable to those observed with DAPA treatment alone. These results support the notion that NF-κB acts as an upstream regulator of the NLRP3 inflammasome and indicate that DAPA may primarily modulate NLRP3 inflammasome activation by suppressing of NF-κB signaling.

Both DAPA monotherapy and combination treatment significantly inhibited CF proliferation while reducing the expression of markers of CF phenotypic transition and collagen synthesis markers. Zhou Lv and colleagues found that NLRP3 inflammasome activation in mouse lung microvascular endothelial cells was associated with increased TGF-β1 expression, which was partially reversed by *Nlrp3* knockdown [45]. TGF-β1 is widely recognized as a key regulator of CF activation, promoting fibroblast proliferation and inducing their transition into an activated myofibroblast phenotype via both Smad-dependent and Smad-independent signaling pathways. In the present study, we similarly observed that in LPS-treated CFs, activation of the NLRP3 inflammasome was accompanied by the upregulation of TGF-β1 expression, enhanced proliferation, increased FAP expression, and elevated collagen synthesis. These changes were significantly attenuated by all three interventions, highlighting the pivotal role of the NLRP3-TGF-β1 axis in fibrosis, although the underlying mechanisms remain unclear. Further studies are required to clarify these underlying pathways.

In summary, this study integrated both *in vivo* and *in vitro* experiments to investigate the regulatory role of DAPA in CFs activation and explore the underlying molecular mechanisms, demonstrating that DAPA suppresses CF activation associated with the NF-κB/NLRP3 pathway inhibition. These findings provide novel insights into the cardioprotective mechanisms of SGLT2is after MI and highlight the synergistic relationship between its anti-inflammatory and anti-fibrotic effects. Additionally, this study achieved dynamic visualization of DAPA’s anti-fibrotic effects in a non-diabetic MI model. The potential application of ^18^F-FAPI PET was preliminarily explored to monitor the therapeutic efficacy of anti-fibrotic treatments, broadening the scope of FAP-targeted molecular imaging for cardiovascular disease diagnosis and treatment.

## 5. Limitations

First, compared to the MI group, the MID group experienced higher mortality during MI induction, primarily due to malignant arrhythmias, reflecting informative missingness. This likely introduced survivorship bias, which might have exaggerated the observed antifibrotic effects. Moreover, the limited sample sizes in some parts of the study might reduce the statistical power and compromise the robustness of the results. Second, this study conducted a preliminary investigation into the effects of DAPA on NLRP3-dependent signaling following MI. Although SGLT2 has been reported to improve mitochondrial dysfunction, whether it regulates NLRP3-dependent signaling through upstream mitochondrial mechanisms remains to be elucidated. In future studies, we will systematically investigate the mitochondria-NLRP3 inflammasome axis using larger sample sizes and *Nlrp3* genetically modified animal models to comprehensively elucidate its molecular mechanisms. Third, this study was conducted in male rats that received a single-dose intervention. Future studies should incorporate female animals, multiple dosing levels, and different administration timing to systematically delineate the sex-, dose-, and time-dependent effects of DAPA. Finally, the findings of this study have not been verified in humans. Existing evidence suggests that the temporal evolution pattern of myocardial FAPI uptake in infarcted hearts differs between rats and humans [46,47]. Further clinical studies are required to validate these potential applications.

## 6. Conclusions

At the organ level, this study revealed that DAPA confers robust anti-fibrotic and cardioprotective effects in a non-diabetic rat model of MI. At the cellular level, DAPA attenuated post-MI fibrosis by inhibiting CF activation, which is associated with suppression of the NF-κB/NLRP3 pathway, thereby improving ventricular remodeling and cardiac function. This study provides novel mechanistic insights into the cardioprotective effects of SGLT2is after MI and offers experimental evidence and mechanistic support for the therapeutic efficacy of targeting NLRP3-mediated inflammation in post-MI myocardial fibrosis.

Moreover, FAP-targeted PET provides a novel molecular imaging modality for the noninvasive visualization and dynamic evaluation of post-MI fibrosis. These results demonstrate that longitudinal ^18^F-FAPI-74 PET imaging enables noninvasive *in vivo *tracking of molecular-level improvements in post-infarction fibrosis following DAPA treatment, providing imaging-based evidence for its inhibitory effects on CF activation. These findings highlight the potential value of FAP-targeted PET for early risk stratification, therapeutic efficacy monitoring, and the precise management of ALVR after MI.

## Data Availability

All data generated or analyzed during this study are obtainable from the corresponding author upon reasonable inquiry.

## Abbreviations

^18^F-FAPI-74, ^18^F-fibroblast activation protein inhibitor-74; ^18^F-FDG, ^18^F-fluorodeoxyglucose; α-SMA, alpha-smooth muscle actin; ALVR, adverse left ventricular remodeling; ASC, apoptosis-associated speck-like protein containing a CARD; bpm, beats per minute; BP, blood pressure; BW, body weight; Casp-1, caspase-1; CMR, cardiac magnetic resonance; CF/CFs, cardiac fibroblast(s); CI/CIs, confidence interval(s); DAPA, dapagliflozin; DBP, diastolic blood pressure; ECG, electrocardiogram; FBG, fasting blood glucose; FOV, field of view; FLASH, fast low-angle shot; h, hours. HF, heart failure; IHC, immunohistochemistry; IL-1β, interleukin-1 beta; IL-18, interleukin-18; LPS, lipopolysaccharide; LAD, left anterior descending; LV, left ventricular; LVEDV, left ventricular end-diastolic volume; LVESV, left ventricular end-systolic volume; LVEF, left ventricular ejection fraction; mean diff., Mean differences; MS, Masson’s trichrome; MI, myocardial infarction; min, minutes; NF-κB, nuclear factor kappa B; NLRP3, NACHT, LRR, and PYD domain-containing protein 3; p-IκB, phospho-IκB; PET, positron emission tomography; p-NF-κB, phospho-NF-κB; ROIs, regions of interest; RT-qPCR, real-time quantitative polymerase chain reaction; SAX, short-axis; T2D, type 2 diabetes; SBP, systolic blood pressure; SGLT2, sodium-glucose transporter 2; SGLT2is, sodium-glucose transporter 2 inhibitors; SUV_max_, maximum standardized uptake value; SUV_mean_, mean standardized uptake value; TGF-β1, transforming growth factor beta 1; TLR4, Toll-like receptor 4; VOI, volume of interest; WB, western blot.
