# Supplementary material for: Dapagliflozin Attenuates Post-Infarction Myocardial Fibrosis Associated With the Inhibition of NLRP3-Dependent Inflammation
Source: Rev Cardiovasc Med. 2026 Oct 20;27(10):51179. doi: 10.31083/RCM51179 (PMC13732652; doi:10.31083/RCM51179)
Supplement: Supplementary file 1 [file 2153-8174-27-10-51179-s1.zip › Supplementary Material.doc]

Supplementary Materials

Dapagliflozin attenuates post-infarction myocardial fibrosis via inhibition of NLRP3-dependent inflammation

Supplementary Table 1. Average weekly food intake per rat.

|  | **Sham** | **ShamD** | **MI** | **MID** | ***p*-value** |
| --- | --- | --- | --- | --- | --- |
| **W1 pre-surgery**  **(g)** | 188.1 ± 19.2 | 191.1 ± 7.6 | 195.9 ± 15.5 | 203.3 ± 10.9 | 0.351 |
| **W1 post-surgery**  **(g)** | 216.8 ± 10.5 | 213.9 ± 11.1 | 211.9 ± 6.4 | 221.2 ± 13.9 | 0.666 |
| **W2 post-surgery**  **(g)** | 232.5 ± 15.3 | 232.4 ± 6.9 | 236.9 ± 9.9 | 219.9 ± 11.2 | 0.226 |
| **W3 post-surgery**  **(g)** | 242.8 ± 13.9 | 245.6 ± 17.7 | 251.9 ± 10.7 | 241.7 ± 5.5 | 0.657 |
| **W4 post-surgery**  **(g)** | 263.9 ± 10.5 | 257.5 ± 8.9 | 257.8 ± 17.7 | 256.5 ± 11.5 | 0.885 |
| **W5 post-surgery**  **(g)** | 270.6 ± 8.3 | 262.3 ± 13.6 | 277.3 ± 16.3 | 270.0 ± 11.9 | 0.548 |
| **W6 post-surgery**  **(g)** | 291.5 ± 13.9 | 284.8 ± 11.8 | 284.2 ± 16.4 | 294.8 ± 10.6 | 0.645 |

Data are shown as means ± SDs. Comparisons among three or more groups: one-way ANOVA with Tukey’s test. Statistical significance: *p* < 0.05.

Supplementary Table 2. Cardiac function parameters at baseline.

|  | **Control group** | **DAPA treatment group** | ***p*-value** |
| --- | --- | --- | --- |
| **LVEDV**  **(mm^3^)** | 282.9 ± 4.0 | 284.8 ± 3.9 | 0.290 |
| **LVESV**  **(mm^3^)** | 85.4 ± 5.1 | 86.2 ± 5.6 | 0.735 |
| **LVEF**  **(%)** | 68.9 ± 1.8 | 69.8 ± 1.7 | 0.263 |
| **LVmass**  **(mg)** | 405.6 ± 12.5 | 400.8 ± 5.8 | 0.280 |

Data are shown as means ± SDs. n = 10 per group. Two-group comparisons: unpaired two-tailed Student’s t-test. Statistical significance: *p* < 0.05.

**Supplementary Fig. 1.** Infarct size comparison between the MI and MID groups (n =10 per group). No significant differences in infarct volume fraction between the two groups. Two-group comparisons: unpaired two-tailed Student’s t-test. Statistical significance: *p* < 0.05.

**Supplementary Fig. 2.** Uncropped original blots corresponding to Figure 4D, Figure 6B/D, and Figure 7C/D. *, LPS stimulation; D6, Dapagliflozin (6 μM); D8, Dapagliflozin (8 μM); D10, Dapagliflozin (10 μM); D+B, Dapagliflozin (10 μM) + BAY 11-7082 (12 μM); D+M, Dapagliflozin (10 μM) + MCC950 (10 μM).
